# Spatial Epidemiology of Recently Acquired HIV Infections across Rural and Urban Areas of North Carolina

**DOI:** 10.1371/journal.pone.0088512

**Published:** 2014-02-10

**Authors:** Margaret Carrel, Joseph J. Eron, Michael Emch, Christopher B. Hurt

**Affiliations:** 1 Department of Geographical & Sustainability Sciences, University of Iowa, Iowa City, Iowa, United States of America; 2 Division of Infectious Diseases, University of North Carolina at Chapel Hill, Chapel Hill, North Carolina, United States of America; 3 Department of Geography, University of North Carolina at Chapel Hill, Chapel Hill, North Carolina, United States of America; National HIV and Retrovirology Laboratories, Canada

## Abstract

Transmission of HIV continues in the United States (US), despite prevention efforts aimed at education and treatment. Concurrently, drug resistance in HIV, particularly in patients being infected with HIV for the first time, poses a threat to the continued success of treatment for HIV positive individuals. In North Carolina, nearly one in five individuals with acute HIV infection (AHI) is infected with a drug-resistant strain, a phenomenon known as transmitted drug resistance (TDR). Few studies of AHI or TDR take into account both the spatial aspects of residence at time of infection and the genetic characteristics of the viruses, and questions remain about how viruses are transmitted across space and the rural-urban divide. Using AHI strains from North Carolina, we examined whether differences exist in the spatial patterns of AHI versus AHI with TDR, as well as whether the genetic characteristics of these HIV infections vary by rural-urban status and across Health Service Areas. The highest amounts of TDR were detected in persons under age 30, African Americans, and men who have sex with men (MSM) - similar to the populations where the highest numbers of AHI without TDR are observed. Nearly a quarter of patients reside in rural areas, and there are no significant differences between rural and urban residence among individuals infected with drug resistant or drug susceptible viruses. We observe similar levels of genetic distance between HIV found in rural and urban areas, indicating that viruses are shared across the rural-urban divide. Genetic differences are observed, however, across Health Service Areas, suggesting that local areas are sites of genetic differentiation in viruses being transmitted to newly infected individuals. These results indicate that future efforts to prevent HIV transmission need to be spatially targeted, focusing on local-level transmission in risky populations, in addition to statewide anti- HIV efforts.

## Introduction

Despite decades of education and prevention efforts, HIV incidence remains relatively stable in the United States (US), at approximately 48,000 new infections each year [1]. However, new HIV infections are not evenly spatially distributed across the US; higher levels of new HIV infections are observed in the Southeast as compared to the rest of the country [1], [2]. Additionally, certain demographic and behavioral risk groups, namely young black men who have sex with men (MSM), have high levels of new HIV acquisition [3]–[6].

Though incidence rates of HIV remain steady in the US, prevalence rates have risen, because people are living longer due in large part to antiretroviral (ARV) therapy. Effective treatment for HIV has led to greatly decreased mortality and increased life expectancy for persons living with the virus in developed countries and resource-limited settings, worldwide [7], [8]. As with other antimicrobials, widespread use of ART has led to the emergence of drug-resistant forms of the virus [9]–[11]. Unlike other pathogens, however, all drug resistance mutations that develop in HIV are “archived” as proviruses throughout the host genome. Thus, once resistance has developed, individual drugs or in some cases entire drug classes may not be useful again for treating the patient[12]. When individuals who are viremic with resistant HIV engage in unprotected sex or share needles, previously uninfected individuals can acquire ARV-resistant HIV, a phenomenon known as transmitted drug resistance (TDR)[13]. The TDR virus may also be passed from one individual to another in the absence of therapy. The prevalence of TDR has remained stable at approximately 10–20% of newly diagnosed individuals in North America[11], [14]–[17] and Europe[18]–[21] and may be higher in acutely infected patients [22]. In the developing world, where treatment options are often limited, the prevalence of TDR appears to be increasing rapidly; 8 years since the widespread rollout of ARVs in east Africa, the prevalence of TDR is nearly 8% [23].

Despite the spatial variation in HIV infection across the US, most investigations of newly acquired HIV and evaluations of TDR versus drug-sensitive viruses in newly infected individuals typically do not take into account geographic location or utilize a coarse spatial resolution such as county or ZIP code. With the collection of HIV genetic sequences from newly infected individuals and sociodemographic information for partner counseling and referral services, new opportunities have developed for the merger of genetics, geography and epidemiology. Spatial molecular epidemiology has the potential to illuminate patterns of HIV transmission, and of TDR variants in particular, indicating populations and places where surveillance and interventions might best be targeted [24]–[26]. In particular, spatial molecular epidemiology can answer questions about whether urban areas act as reservoirs for rural HIV infection, or whether circulating strains of HIV differ between rural and urban areas.

In the present study of acutely HIV-infected individuals in North Carolina (NC), we had three specific aims. First, we wished to determine the geographic and genetic distributions of new HIV cases across NC – including the subset with TDR. Second, we sought to understand whether genetic relatedness among all viruses and viruses from specific epidemiologic subgroups was associated with residential location characteristics, such as urban or rural status and health service region, as well as the geographic distance between individuals. Finally, we wanted to examine whether TDR viruses exhibited different spatial and genetic patterns than did non-TDR, drug-susceptible (DS) viruses.

## Data and Methods

### Ethics Statement

All participants in the Duke/UNC Acute HIV Consortium Database project provided informed consent for their de-identified sociodemographic, immunological, and virological information to be used for research purposes. The Institutional Review Boards at the University of North Carolina at Chapel Hill and the University of Iowa reviewed and approved the protocol of the study described herein.

### Study population

All subjects included in this study were diagnosed with seronegative acute HIV infection (AHI) between 1998–2009 and enrolled in the Duke/UNC Acute HIV Consortium Database project [27]. Detailed descriptions of the case definition and database have been published previously [28]–[30]. In brief, subjects in the database were referred to one of the participating institutions either by community medical providers or the Screening and Tracing Active Transmission (STAT) program of the NC Department of Health and Human Services (NC-DHHS). In STAT, any individual presenting to a publicly funded HIV or sexually transmitted infection testing site who has blood drawn for HIV antibody or syphilis testing is also tested for HIV nucleic acids. HIV RNA is detectable approximately 10–14 days earlier than antibodies against HIV, allowing identification of very early infections [31]. RNA-positive, antibody-negative patients are referred for further evaluation; a majority provide consent for their data to be included in the Consortium database.

### Genotypic resistance testing

From the earliest possible plasma sample for each subject, the *pol* region of the HIV-1 genome was sequenced using primers spanning all of the protease and the majority of the reverse transcriptase gene (from codons 1-100 and 38-250, respectively). Genosure (Laboratory Corporation of America, Research Triangle Park, NC, USA) or Trugene (Siemens Healthcare Diagnostics, Tarrytown, NY, USA) primers were used for all sequencing analyses. Raw nucleotide sequence data were analyzed with the Stanford University HIV Drug Resistance Database (http://hivdb.stanford.edu) to characterize ARV-resistant viruses; relevant mutations were defined by the 2009 World Health Organization list of surveillance drug resistance mutations (SDRMs)[10].

### Phylogenetic analyses

Sequences were aligned using Multiple Sequence Comparison by Log-Expectation (MUSCLE) [32], then edited manually using Se-Al v2.0a11 (Andrew Rambaut, University of Edinburgh). The final data included codon positions 4-98 of protease and 38-240 of reverse transcriptase, based on numbering from an HXB2 consensus sequence (NCBI Reference Sequence NC_001802.1). A maximum likelihood (ML) tree was inferred in RAxML v7.2.0 [33], under a generalized time-reversible (GTR) model of nucleotide substitution with 1000 bootstrapped replicates. A matrix of genetic distances separating all pairs of taxa from the solved, consensus ML tree were extracted from a Newick tree file using PATRISTIC [34], with results exported as a comma-separated file for manipulation and analysis.

### Geocoding

NC-DHHS Communicable Disease Branch personnel conducted a special query to retrieve addresses for each AHI case at the time of diagnosis. Those addresses were geocoded (assigned latitude and longitude), and this coordinate location was then matched to the 2000 Census block group (CBG) in which it was located. The centroid of the CBG was then calculated and the latitude/longitude of this anonymized point was assigned to each AHI case. Individual addresses were then deleted from the database. The CBG is the smallest unit for which demographic data collected through the Census are publicly available; each contains approximately 600–3000 individuals, and they vary in geographic size. Once the assignment to a CBG was completed, all individual address and geocoded information was deleted. This geocoding and linkage to CBG of residence at time of infection was done prior to linking to clinical and virological information from the Consortium database. Because only the CBG number and the coordinates of its centroid were recorded in the final data set, patient privacy was maintained at all times.

Cases were mapped according to their CBG of incidence and stratified by demographic and behavioral variables and drug resistance status. To investigate the potential association between residential location and genetic relatedness of viruses, cases were classified as either rural or urban according to the designations created in the 2000 Census, with 17 urbanized areas and 90 urban clusters defined in NC. Urbanized areas and urban clusters were collapsed into one “urban” category for the purposes of this study. Any case who's CBG fell outside the bounds of an urban area were classified as rural. To further investigate potential spatial differentiation in genetic patterns of viruses, cases were assigned to the Health Service Area (HSA) of residential location at time of diagnosis. There are six HSAs in North Carolina, each of which represents a set of contiguous counties that are used in the planning of health care provision across the state.

To complement the pair-wise genetic distance matrix, a pair-wise geographic distance matrix was generated, indicating the distance in kilometers between each AHI case in the dataset.

### Statistical Methods

Characteristics for all AHI patients, stratified by TDR status, were summarized using descriptive statistics. We assessed the relationship between demographic and behavioral characteristics and the presence of TDR using Pearson's χ^2^ test. Exact probability values were calculated, given the overall low numbers of observations within categories. Pearson's χ^2^ test was also used to measure differences in rural/urban categorization by demographic and behavioral characteristics, as well as HSA of residence. Statistical significance was set at *P*<0.05 for all analyses.

Scatterplots of pair-wise genetic versus geographic distances were generated, stratifying observations by TDR status, as well as HSA and rural/urban status. Pair-wise genetic distances were also stratified according to whether the pair was a rural-rural set, a rural-urban set or an urban-urban set, in order to understand differences in genetic relationships according to urbanicity. The same was done with HSA location of incidence, to explore inter-HSA and intra-HSA differences in genetic distances among viruses.

All geocoding and mapping was conducted in ArcGIS 10.0 (Esri, Redlands, CA). Chi-square tests were calculated in SAS 9.3 (SAS Institute, Inc., Cary, NC). Plotting was conducted in R using the *ecodist* package (R Foundation for Statistical Computing, Vienna, Austria). Median genetic values were calculated for lower triangle matrices using R and the difference in median genetic values were assessed using Wilcoxon tests for bivariate groupings and Kruskal-Wallis tests for trivariate groupings, also in R.

## Results

We successfully geocoded 81 of 143 AHI cases identified between1998–2009 (57%); 17 (21%) had ≥1 mutation indicating TDR. Three AHI cases without TDR and 4 cases with TDR lacked demographic and behavioral information. Among the remaining 74 cases, the median age of patients with drug-susceptible (DS) virus was 26 years (interquartile range [IQR], 21–36; Table 1), while the median age of TDR cases was 23 (IQR, 21–27). Ninety-three percent of all DS AHI cases were among men (n = 57), with a similar proportion observed among subjects with TDR (85%, n = 11). The majority of AHI cases were Black men, 52% with DS virus and 46% with TDR. Of those Black men, the majority (74% of DS and 83% of TDR) were MSM. These proportions reflect the disproportionate burden of HIV in the Southeastern US[35]– especially among young Black MSM[5].

**Table 1:** Demographic and behavioral characteristics of AHI cases, stratified by drug sensitivity.

Drug Susceptible Cases (n = 61)		TDR Cases (n = 13)		Chi-square	Exact p-value
	Median (IQR)		Median (IQR)		
**Age**	26 (21–36)	**Age**	23 (21–27)	7.586	0.101
	n (%)		n (%)		
**Sex**		**Sex**		1.121	0.582
Male	57 (93.4%)	Male	11 (84.6%)		
Female	4 (6.6%)	Female	2 (15.4%)		
**Race/Ethnicity**		**Race/Ethnicity**			
White	21 (34.4%)	White	4 (30.8%)		
Black	34 (55.7%)	Black	7 (53.8%)		
Hispanic	6 (9.8%)	Hispanic	1 (7.7%)		
		Asian/Pacific Islander	1 (7.7%)		
**Mode**		**Mode**		1.107	0.879
MSM	42 (68.9%)	MSM	9 (69.2%)		
Heterosexual	14 (23%)	Heterosexual	4 (30.8%)		
Sex Partner at Risk	2 (3.3%)				
STI Diagnosis	1 (1.6%)				
No Acknowledged Risk	1 (1.6%)				
None listed	1 (1.6%)				
**Location**		**Location**		0.574	0.542
Urban	47 (73.4%)	Urban	14 (82.4%)		
Rural	17 (26.6%)	Rural	3 (17.6%)		
**HSA**		**HSA**		9.594	0.081
1	6 (9.4%)	1	0 (0%)		
2	7 (10.9%)	2	6 (35.3%)		
3	5 (7.8%)	3	2 (11.8%)		
4	26 (40.6%)	4	7 (41.1%)		
5	10 (15.6%)	5	2 (11.8%)		
6	10 (15.6%)	6	0 (0%)		

Note that three drug susceptible and four drug resistant cases had no demographic and behavioral information available.

No statistically significant differences with respect to age, sex, race/ethnicity, mode of acquisition or rural/urban residence were observed between individuals with TDR and those with DS viruses (Table 1). There was a trend toward variation in TDR status by HSA (*P* = 0.08).

**Figure 1:**
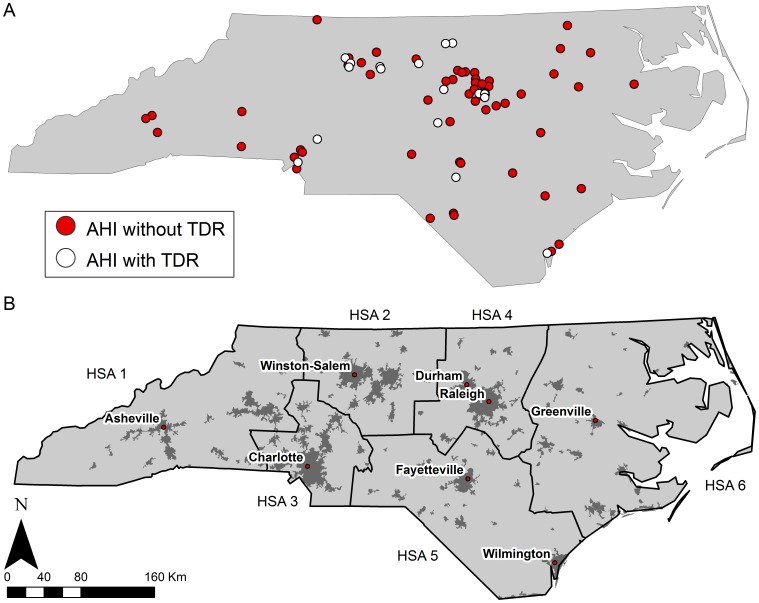
Spatial distribution of AHI and TDR positive census block groups (A) and Census-designated urban areas and major urban locations in North Carolina, with HSA boundaries (B).

Fifty-four individuals live in urban areas and 20 in rural areas. We observed no significant differences in the distribution of urban versus rural-dwelling patients, when categorized by race/ethnicity or mode of acquisition (MSM versus heterosexually acquired; Table 2). However, when we categorized patients by their HSA of residence, there was a statistically significant difference in rural/urban status; larger proportions lived in urban settings in all HSAs except HSA 6, which encompasses most of the northeastern portion of the state (*P* = 0.008; Figure 1B).

**Table 2:** Urban/rural status of cases by race/ethnicity, risk groups and HSA designations.

	Urban	Rural	Chi-square	Exact p-value
**Race/Ethnicity**			4.1328	0.263
White	19	6		
Black	27	14		
Hispanic	7	0		
Asian	1	0		
**Mode**				
MSM	39	12	0.663	0.533
Black MSM	21	7	0.375	0.738
Young Black MSM	20	5	0.977	0.48
Heterosexual	12	6		
**HSA**			15.299	0.008
1	4	2		
2	12	1		
3	5	2		
4	26	7		
5	11	1		
6	3	7		

**Figure 2:**
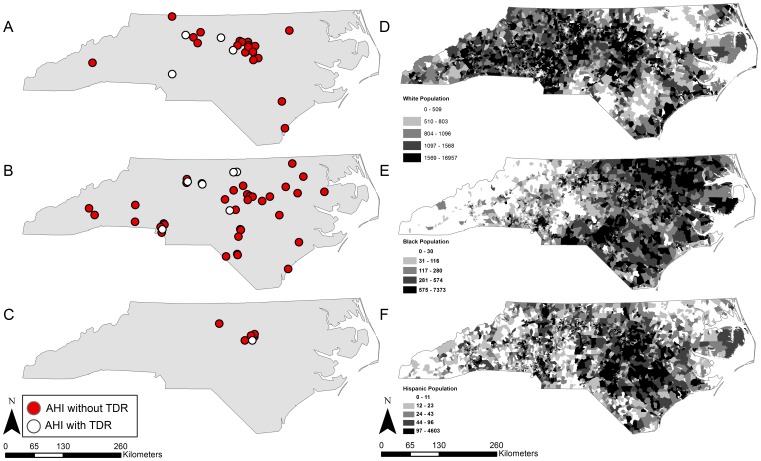
Distribution of AHI and TDR according to the most frequently reported race/ethnicities in the dataset (A: White, B: Black, C: Hispanic) and number of each race/ethnicity reported in block groups in the 2010 Census (D: White, E: Black, F: Hispanic).

**Figure 3:**
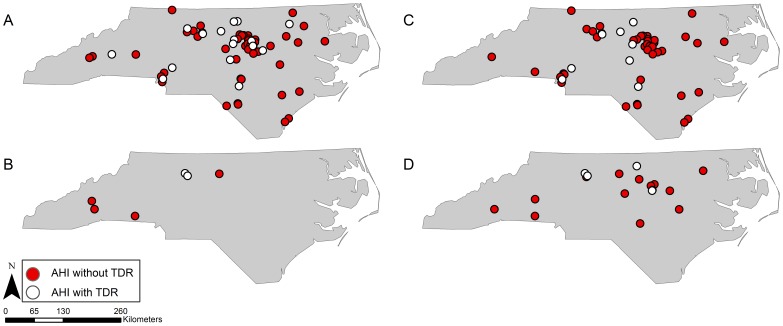
Distribution of AHI and TDR cases by sex (A: men, B: women) and by mode of acquisition(C: men who have sex with men (MSM), D: heterosexual intercourse).

Maps of AHI and TDR cases reveal several noteworthy patterns. First, though the distribution of cases reflects major population centers in NC, not all cases were exclusive to urban areas (Figure 1). This is especially true for the rural and economically disadvantaged eastern portion of the state (HSA 6) and the southern coastal plain (HSA 5). Second, cases among Whites and Latinos conformed to what would be expected statistically based on subpopulation density, but Black subjects were more evenly distributed geographically, including the western mountainous region where far fewer Black North Carolinians reside (Figure 2). Cases among women seemed more concentrated in the central (Piedmont) region and the mountain west, while male cases were widespread across the entire state (Figure 3A & 3B). Finally, AHI cases among MSM were observed in both urban centers and more rural areas, widely distributed across NC. In contrast, cases of AHI among individuals reporting heterosexual sex as their HIV risk behavior were more often located in or around the central Piedmont area (Figure 3C & 3D).

**Figure 4:**
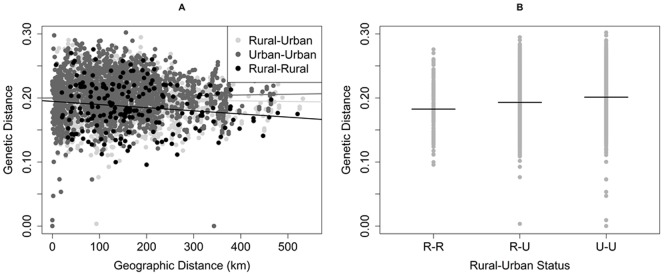
Exploring Rural/Urban genetic variation. (A) Pair-wise genetic versus geographic distance for cases, classified as Rural-Urban, Urban-Urban or Rural-Rural based on CBG of patient at time of infection. (B) Genetic distances for all viruses, stratified by Rural/Urban pair relationship with median value indicated by a bar. R-R indicates both cases are from rural areas, R-U indicates one rural and one urban case, and U-U indicates both cases are from urban areas.

Viruses from rural dwellers were more closely related to one another than they were to viruses from urban areas. Scatterplots of pairwise genetic distance versus pairwise geographic distance between cases, categorized according to rural or urban residence at time of infection, indicate that viruses sampled from rural residents were separated by smaller pairwise genetic distances, even across geographic space, than viruses found in urban residents (urban-urban, median = 0.201; Figure 4A & 4B). The same was true when we examined pairings of rural viruses with urban ones; the genetic distance between pairs of rural viruses (median = 0.183) was smaller than the distance separating rural viruses from urban ones (rural-urban, median = 0.193).

**Figure 5:**
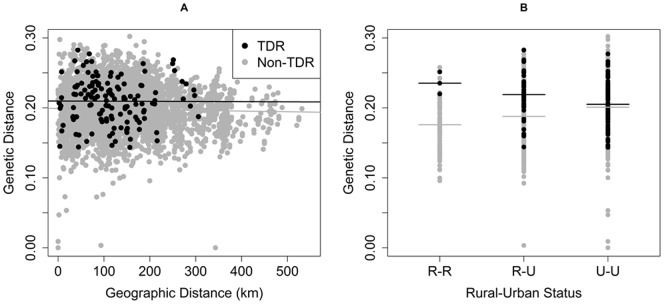
Exploring TDR versus non-TDR genetic and geographic variation. (A) Pair-wise genetic versus geographic distance for TDR and non-TDR viruses, and (B) TDR and non-TDR genetic distances by Rural/Urban pair relationship with median value indicated by a bar.

In a similar analysis of DS viruses and those with TDR, clustering was observed among TDR viruses at lower geographic distances, indicating that DS viruses were more widely distributed across the state (Figure 5A). Despite the smaller geographic range among TDR viruses, the pairwise genetic distances of TDR and DS viruses share the same general pattern over geographic space, fluctuating around 0.20.

When the various pairwise genetic distances separating TDR viruses from one another and DS viruses from one another were stratified according to residence at the time of diagnosis (rural versus urban), a different pattern emerged (Figure 5B). Among DS viruses and among TDR viruses, the range of genetic distances for urban-urban pairings (DS median = 0.201, TDR median = 0.205) was similar; Wilcoxon tests indicated no statistically significant variation between TDR and DS genetic distances among urban-urban (p = 0.37) case pairs. In contrast, genetic distances among rural-urban case pairs were significantly different for TDR versus DS viruses (median = 0.219 & 0.188 respectively, Wilcoxon p<0.01). TDR viruses identified in rural residents were not genetically similar (median genetic distance = 0.235) and had greater genetic distance than did DS rural-rural viruses (median = 0.176). Wilcoxon tests indicated a statistically significant difference in TDR versus DS viruses in rural-rural case pairs (p = 0.008).

**Figure 6:**
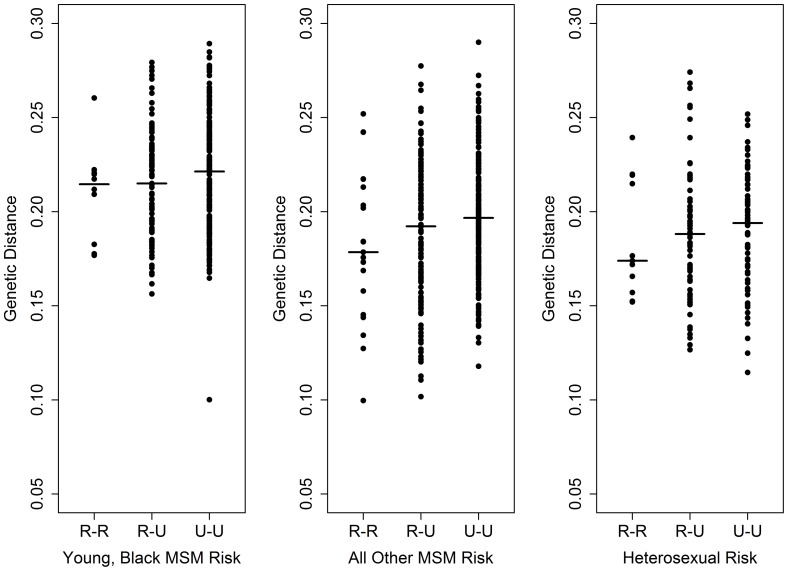
Viral pair-wise genetic distances among young, black MSM risk patients, among other MSM patients, and among heterosexual risk patients, stratified by Rural/Urban pair relationship. Median genetic distance for each group is indicated by a bar.

We then assessed the genetic relatedness between pairs of viruses from young, Black MSM, all other MSM, and heterosexuals, categorized into rural-rural, rural-urban, and urban-urban groupings (Figure 6). Young, Black MSM had narrower ranges of pairwise genetic distances than all other MSM and heterosexuals, across rural-urban categories – but young, Black MSM viruses also exhibited the highest degree of genetic distance. The median pairwise genetic distances for young, Black MSM were 0.215, 0.215 and 0.221 for rural-rural, rural-urban, and urban-urban categories respectively, which were higher than the values for all other MSM (0.179, 0.192 and 0.197; Kruskal-Wallis P<0.01) and heterosexuals (0.174, 0.188, and 0.194; Kruskal-Wallis P<0.01).

**Figure 7:**
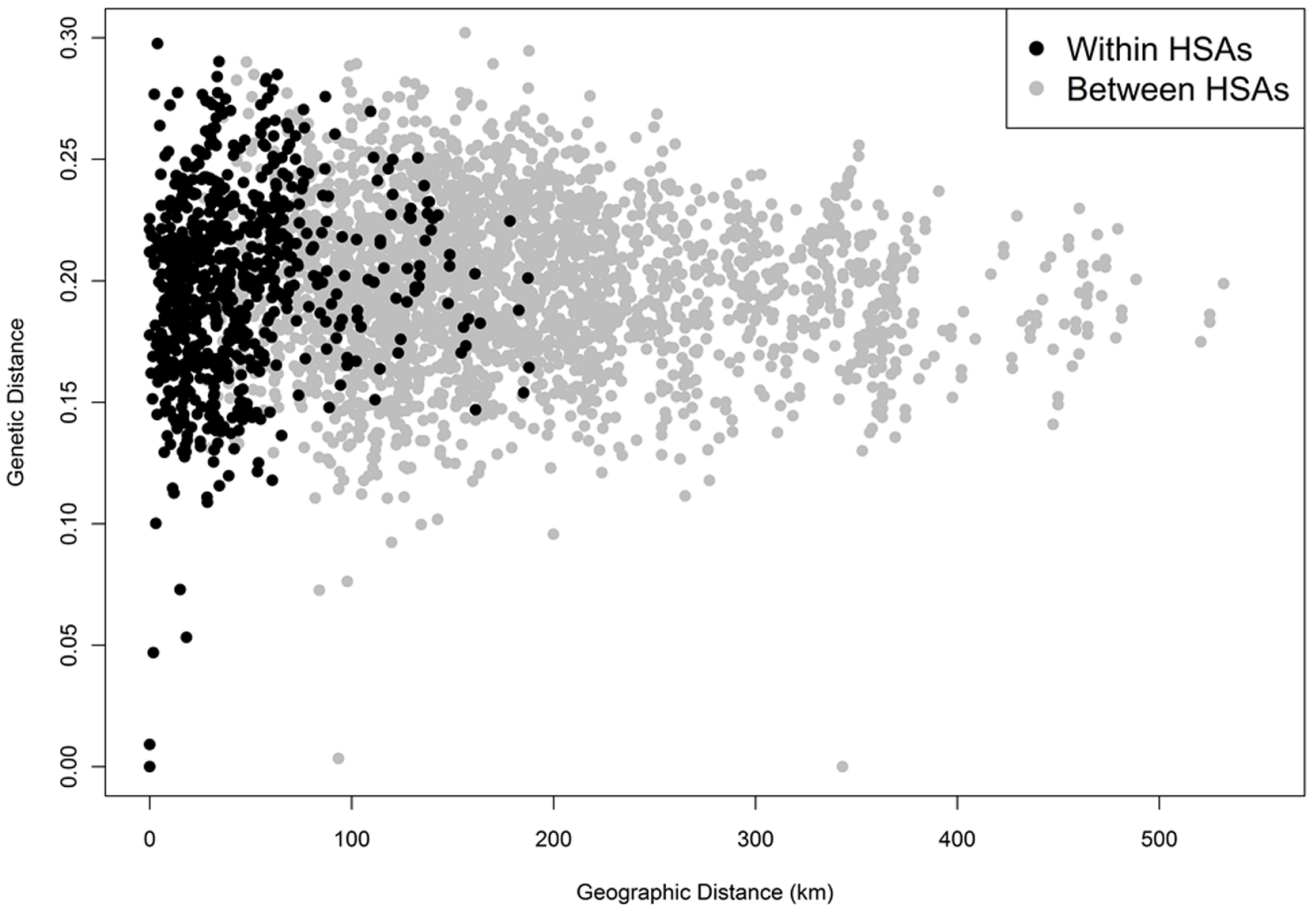
Pair-wise genetic versus geographic distance for cases located in the same HSA (black) or different HSAs (grey).

**Figure 8:**
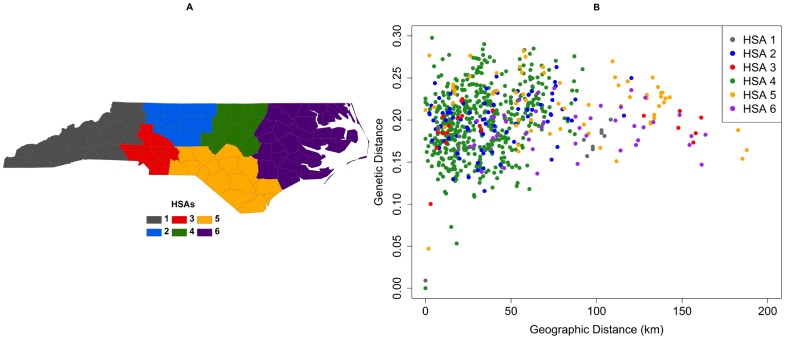
Pair-wise genetic versus geographic distance within HSAs.

Viruses from individuals living in the same HSA had the same general distribution of pairwise genetic distances as did persons living in different HSAs, often hundreds of kilometers away from one another (Figure 7). The level of genetic dissimilarity is fairly constant across the state, although the genetic distance declined slightly as the geographic distance separating the patients increased. Plotting intra-HSA genetic variation by individual HSAs of incidence (i.e. HSA 1 versus HSA 1 viruses) revealed HSA-specific genetic patterns (Figure 8). The majority of viruses in the dataset were found in individuals residing in HSA 4 (Figure 8B & Table 1), shown in green. These viruses exhibited a narrower geographic range than did viruses in other HSAs; HSA 4 is one of the smaller HSAs in the state. Despite the smaller geographic range, there is a high level of genetic distance between viruses in HSA 4. In contrast, HSA 6 viruses are widely spaced geographically but exhibit low inter-virus genetic distance.

**Figure 9:**
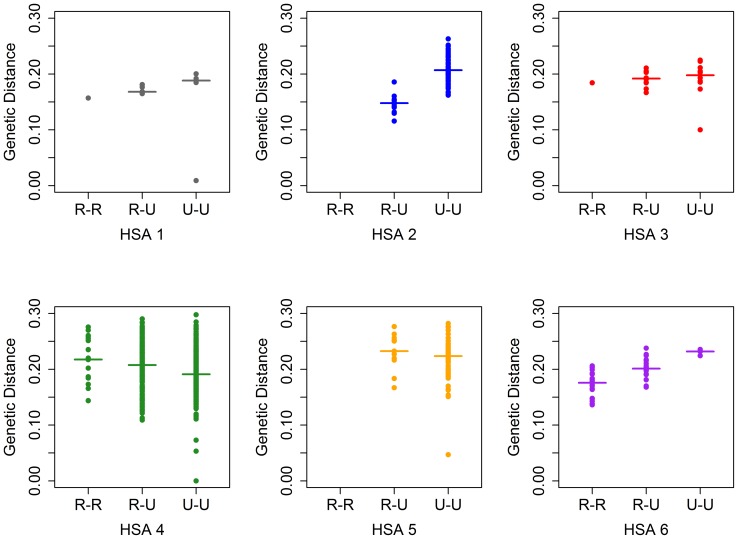
Pair-wise genetic distances for AHI cases, stratified by rural/urban status. Median genetic distance is indicated by a bar. HSAs 2 and 5 had only one rural case, so had no R-R genetic pairs.

To explore whether these differences in genetics by HSA of residence were driven primarily by rural or urban residential location, intra-HSA genetic distances between viruses were categorized by rural-rural, rural-urban and urban-urban pairings (Figure 9). Urban-urban genetic relatedness across all HSAs varied; some viruses were closely related and other had a high degree of genetic distance. HSAs 2 and 5 had only one rural sample, so had no rural-rural pairs. Rural-rural viruses in HSA 4 have high genetic distance, as high as do rural-urban and urban-urban virus pairs. In contrast, rural-rural viruses in HSA 6 have much lower genetic distances than do rural-urban and urban-urban viruses in that region. Kruskal-Wallis tests indicated significant variation in genetic distances across rural-rural, rural-urban and urban-urban case pairs within HSAs 2 (p<0.001), 4 (p<0.001) and 6 (p<0.001). Genetic distances across these residential designations in HSAs 1 (p = 0.067), 3 (p = 0.562) and 5 (0.225) did not vary significantly.

## Discussion

This is the first study of the spatial epidemiology of recently acquired HIV infection in the United States. We find some evidence for geographic restriction of DS and TDR viruses in rural versus urban areas. As in the rest of the United States, North Carolina's HIV epidemic is principally among young, Black MSM. There were no significant differences in individual-level characteristics between rural and urban residence, suggesting that the epidemic is impacting this key risk group uniformly across the state, regardless of where these men live. Furthermore, nearly one quarter of the individuals in the sample lived in rural areas, highlighting the importance of maintaining access to HIV testing and treatment resources in less urbanized areas of the state.

The pairwise genetic distances separating viruses from rural dwellers were smaller than those separating viruses from urbanites, and these distances decreased slightly as geographic distance between patients widened. This suggests that, to some degree, there are separate sub-epidemics occurring simultaneously in the state, with transmissions occurring with some viruses among rural residents and other, genetically unique viruses among urban dwellers. The best evidence for this phenomenon came from our analysis of viruses from each of the six HSAs across the state. In HSAs 1 (mountains), 3 (Charlotte metropolitan area), 4 (Raleigh-Durham), and 5 (Fayetteville to Wilmington), we observed pairwise genetic distances toward the lower end of the range within urban-urban pairs, while HSA 6, the rural and economically depressed Eastern region of the state, had the lowest genetic distances separating rural-rural pairs. In fact, the median distance among these rural pairs in HSA 6 was the lowest across all rural/urban categories in all HSAs, potentially indicating a tighter network of transmission in which viruses were highly genetically related. Further investigation with a larger sample from HSA 6 would allow this to be more definitively assessed.

With respect to TDR versus DS viruses, our analyses indicated that HIV isolates with primary resistance seemed to be more geographically restricted, and were more centered in urban areas. Though the presence or absence of drug resistance was not associated with any individual-level characteristics, this urban/rural dichotomy suggests that transmission of resistant HIV may be more likely in urban areas, which historically have been home to more HIV treatment and care resources. In the early days of the epidemic, many patients relocated to the areas closest to treatment centers, to facilitate frequent trips to providers for clinical care. It is therefore plausible that focally greater densities of patients with HIV treatment experience might sustain more frequent detection of TDR among newly diagnosed patients.

In understanding the potential for urban areas to act as reservoirs of infection for rural areas, several findings suggest mixing of viruses between these categories across the state and a close relationship between urban and rural HIV genetics. Similar genetic distance distributions for intra-HSA and inter-HSA case pairs suggests that there is inter-regional mixing of viruses across the state, and the fact that viruses found in individuals residing far apart from one another are genetically similar suggests that spatial distance is not a barrier to genetic similarity. Similar findings have been observed in Mississippi, where viruses clustered genetically were not clustered geographically [25]. Urban areas of North Carolina have high degrees of genetic distance at small spatial scales, typically higher than the genetic distance found in rural areas or across the rural-urban divided, as seen in Figures 6 & 9. These higher amounts of genetic differentiation could indicate either higher levels of transmission, resulting in greater amounts of genetic variation, or higher levels of introduction of new viral variants into urban areasfrom other sexual networks. Uniquely in the dataset, however, HSA 4 had genetic distances in rural-rural virus pairs that were higher than the levels of genetic distances in rural-urban and urban-urban virus pairs in the same regions, suggesting either that rural residents of this HSA are acquiring a variety of viruses from urban areas within the region or that there is high genetic diversity in viruses circulating in rural areas. In contrast, viruses found in HSA 6 rural resident case pairs had low amounts of genetic distance. This again highlights the possibility that rural residents in HSA 6 are all acquiring infection in the same places or from the same sexual networks, resulting in high genetic similarity.

Perhaps the most important limitation of our study is the sample size. While small numbers of observations are not uncommon in genetic studies of sensitive diseases, caution is still warranted in generalizing our results to larger populations or different geographic settings. For instance, compared to chronically infected patients entering our HIV clinical cohort based in central North Carolina, the recently infected individuals studied here are younger and more likely to be black and endorse sex with men[36]. Additionally, a variety of approaches exist for reconstruction of phylogenetic trees – and thus the estimated genetic distances between taxa may vary somewhat depending on the model of nucleotide substitution and the computational method used.

The merging of spatial analysis, phylogenetic methods and epidemiology holds potential for understanding how and why infectious diseases evolve over space and time. This capability, however, is often hampered by a lack of spatial attributes collected for places of infection. Additionally, for highly sensitive diseases such as HIV, the ability to access datasets containing such information is frequently limited because of privacy concerns. While assigning cases to the Census block group is problematic for finer scale analysis, it is sufficient for this descriptive analysis while protecting the identities of individuals. Collaborations between geographers, epidemiologists and state health agencies can use such aggregation or other spatial offset techniques to enable critical questions regarding the spatial epidemiology of infectious diseases to be answered without compromising patient confidentiality.

Results of this study indicate that there is no strong distinction between rural and urban HIV genetics, or between rural and urban acquisition of TDR, but that the relationship between rural and urban residential status and HIV varies across the state. Understanding how HIV is shared between urban and rural populations, particularly among young black MSM, the predominant group in the study, is crucial to our ability to limit new HIV infections.
